# Patient-reported outcomes and complications of a new-generation total knee system: a randomized controlled trial

**DOI:** 10.2340/17453674.2025.43004

**Published:** 2025-02-25

**Authors:** Kristian R L MORTENSEN, Lina H INGELSRUD, Anders ODGAARD, Andreas KAPPEL, Claus VARNUM, Henrik SCHRØDER, Kirill GROMOV, Anders TROELSEN

**Affiliations:** 1 Department of Orthopedic Surgery, Copenhagen University Hospital Hvidovre; 2 Department of Orthopedic Surgery, Copenhagen University Hospital Gentofte; 3 Department of Orthopedic Surgery, Copenhagen University Hospital Rigshospitalet; 4 Department of Clinical Medicine, University of Copenhagen; 5 Interdisciplinary Orthopaedics, Department of Orthopedic Surgery, Aalborg University Hospital; 6 Department of Orthopedic Surgery, Lillebælt Sygehus – Vejle; 7 Department of Orthopedic Surgery, Næstved Sygehus, Denmark

## Abstract

**Background and purpose:**

Documentation of new-generation implants’ short-term performance could reassure surgeons and patients about their use, while awaiting the long-term outcome. Our aim was to compare the performance of a newer total knee arthroplasty (TKA) system with its predecessor, measured by patient-reported knee function, pain, and complication rate.

**Methods:**

We performed a multi-center, randomized, controlled trial (clinicaltrials.gov ID: NCT03073941). 314 patients with primary osteoarthritis were randomized to treatment with a Persona or NexGen cruciate-retaining TKA system and followed for 2 years. The primary outcome was measured with the patient-reported outcome (PRO) Oxford Knee Score (OKS) 2 years post-surgery. Secondary outcomes were the OKS-Activity and Participation questionnaire (OKS-APQ), Forgotten Joint Score (FJS), EQ-5D-3L, and number of complications during the study period. Responder analyses were performed using Patient Acceptable Symptom State (PASS) and Minimal Important Change (MIC) criteria.

**Results:**

Primary outcome was available from 289 patients (92%). We found no difference in adjusted mean OKS between the groups 2 years post-surgery (0.1, 95% confidence interval –1.4 to 1.7). We found no significant differences in adjusted mean of secondary PROs, PRO time-weighted averages, proportion of patients with PASS or MIC, or complications 2 years post-surgery.

**Conclusion:**

We found no difference in OKS 2 years post-surgery, or in any secondary variables analyzed including complications, between the 2 TKA systems. Short-term safety and performance of the Persona TKA was comparable to its predecessor

Treatment with total knee arthroplasty (TKA) is a common surgical treatment for end-stage knee osteoarthritis, and it is widely accepted as a cost-effective and successful treatment [1]. Despite this, 10–20% of patients remain unsatisfied after TKA [1,2]. The effort to improve treatment for the group of unsatisfied patients has led manufacturers to continuously optimize the design of TKA implants, resulting in new designs introduced to the market frequently. However, new designs are often introduced with limited documentation of performance and safety [3,4], although evidence on early performance could support patients, surgeons, and hospitals decision on which implant to use.

The Persona Total Knee System (Zimmer-Biomet, Warsaw, IN, USA) was introduced in 2012 as successor of the NexGen Total Knee System. Persona features an anatomically shaped tibial baseplate, with finer increments in polyethylene thickness and femoral component sizes, including a standard and a narrow femoral profile. These design features are intended to improve performance, as matching the bony anatomy and reconstructing ligament tension may be done more accurately [5]. The surgical learning curve, early revision rates, fixation, and patient-perceived treatment outcome using this system have been documented through observational study designs, and one randomized controlled trial (RCT) [6-9]. However, a large-scale comparison of early clinical outcome of treatment with the new system is warranted, to establish whether the proposed enhancement of the TKA system, regarding the matching of bony anatomy and reconstructing accurate ligament tension, results in superior patient-perceived treatment outcome compared with the previous generation TKA system.

There is a growing focus on evaluating patient satisfaction, which is a multifactorial aspect. This has been underlined in a recent study, where persistent pain and functional limitations were more important reasons for patient dissatisfaction than reoperations after total joint arthroplasty [10]. Such findings emphasize the need for short-term evaluation of implants from a patient’s perspective. While awaiting survival results of the implants after 10 and 15 years, these studies can aid our understanding of some aspects of treatment quality when patients are expected to have returned to full function after surgery. Therefore, we designed a 2-arm superiority RCT to assess whether the clinical performance and safety of the Persona Total Knee System lead to improved patient outcomes compared with its predecessor. Our primary aim was to compare patient-perceived knee function and pain, measured with the Oxford Knee Score (OKS), 2 years post-surgery. Our secondary aims were to compare activity and participation, joint awareness, health-related quality of life, and number of complications between the implant options during the first 2 years post-surgery.

## Methods

### Study design

We performed a multicenter, single-blinded, superiority RCT where we randomized 314 patients to treatment with either the newer TKA system or its predecessor. Results were reported in accordance with the CONSORT statement and the CONSORT PRO extension [11].

### Setting

The 314 patients were recruited from the orthopedic outpatient clinic of 5 different hospitals in Denmark: Hvidovre, Gentofte, Farsø, Vejle, and Næstved. Our inclusion period lasted from November 28, 2016 until November 19, 2019 and participants were followed for 2 years post TKA surgery, with follow-up visits at 3 months, 1, and 2 years post-surgery.

Screening was done by the surgeons. Inclusion criteria were: indication for primary TKA due to clinically and radiologically verified osteoarthritis (OA). We used the Kellgren–Lawrence classification to evaluate OA severity.

Our exclusion criteria were: patients who were below 18 years of age, unable to understand Danish, had severe comorbidities (ASA > 3), were not clinically suited to receive a cruciate-retaining (CR) implant, not expected to complete all follow-up visits, had terminal illness, rheumatoid arthritis, or traumatic etiology of the OA, had prior surgeries to the affected knee with either osteosynthesis or surgery to the ligaments (including ACL or PCL), had alteration in pain perception caused by diabetes or other comorbidity, and if the patient was deemed unsuitable for CR implants during surgery.

### Randomization

Once informed, all participants signed written consent to study participation prior to inclusion. The treatments were allocated randomly, using a 1:1 computer-generated allocation sequence, which was concealed in non-transparent envelopes. The envelopes were produced and sealed centrally by the study sponsor. At the beginning of the study, 2 blocks of 16 envelopes were dispatched to the hospitals. Enrollment was competitive from here onward, and hospitals received blocks of 16 as required. As enrollment came close to fulfillment, blocks of 4 and 2 would be dispatched to the hospitals.

The envelopes would be opened and inspected by the surgeon performing the surgery on the day of surgery, with the patients still being blinded until after the 2-year follow-up visit.

### Surgical procedure

The treatment allocations were either the Persona or NexGen TKA system. Both implant types were implanted with a cemented tibial component, and a CR bearing was used. 1 case was exceptionally operated with an ultra-congruent bearing (Persona). All Persona femoral components were cemented. In the NexGen group, we used primarily cemented CR femoral components. However, to best accommodate the surgeon’s choice for each patient, 24 femoral components in the NexGen group were uncemented. In the Persona group, all femoral components were non-coated. All bearings used in both groups were made from ultra-high molecular weight polyethylene.

TKA was performed by senior surgeons, with 1 assistant. All surgeries were performed according to the manufactures’ technical guide to the allocated implant. The surgical procedure was performed through a medial parapatellar approach and with a target of mechanical, neutral alignment with predefined boney cuts and ligament releases as needed. The bony cuts were performed using an extramedullary tibia guide and an intramedullary guide on the femur. We resurfaced the patella in all cases, and no drain was used. All patients followed a well-described fast-track setup post-surgery, with early mobilization, standard pain treatment, and rehabilitation until discharge [12].

### Outcome measures

Patient-reported outcomes (PROs) were used to measure the performance of each treatment option, and the Oxford Knee Score (OKS) 2 years post-surgery was the primary outcome in this study. OKS was developed to assess patient-perceived outcome of TKA, and contains 12 items that score patients’ knee function and pain [13]. Each item is scored from 0 (worst) to 4 (best), which then accumulates into a total score, ranging from 0 (worst) to 48 (best). Good evidence of OKS’s psychometric properties, such as content and construct validity, reproducibility, and responsiveness, has been established in previous studies [14].

In addition to the OKS, patients’ activity, awareness of knee joint, and health-related quality of life were measured through PROs. Oxford Knee Score Activity & Participation (OKS-APQ) is an 8-item questionnaire that assesses higher level of patients activity and participation and is scored from 0 (worst) to 100 (best) [15]. The Forgotten Joint Score (FJS) is a 12-item questionnaire that measures patients’ joint awareness after TKA and is scored from 0 (highly aware of knee joint) to 100 (not aware of knee joint) [16]. The EQ-5D-3L [17] is a descriptive system that evaluates patients’ self-perceived health-related quality of life in 5 dimensions, which summarizes into an index score ranging from –0.624 (worst) to 1 (best) [18]. The EQ-5D-3L additionally scores a VAS, where patients are asked to evaluate their overall health status from 0 (worst) to 100 (best). PROs were answered at all follow-up visits. All PROs were collected on paper at follow-up visits. In cases where patients did not attend in person for follow-up visit, PROs were forwarded via regular mail or collected via phone.

PROs were additionally compared with a time-weighted average (TWA) approach, which is the area under curve of patients’ PRO progressions normalized to the total observation time [19]. This allowed for comparison of patients with different observation periods caused by, e.g., loss to follow-up before primary end point at 2 years. As such, patients with any postoperative PROs available were included in the analysis.

OKS and FJS were further analyzed by assessing the proportion of patients reaching the responder criteria of patient acceptable symptom state (PASS) and having a minimal important change (MIC). The PASS concept was developed as a cut-off value in OKS for which patients scoring above that cut-off would describe their knee symptoms as “satisfactory” 1 and 2 years after TKA [20]. PASS would be considered for patients with an OKS ≥ 27 at 3 months and OKS ≥ 30 1 and 2 years post-surgery [20]. MIC represents the minimal change in OKS or FJS that an average patient would consider important. We considered cases with an increase in OKS ≥ 8 and in FJS ≥ 14 to have achieved MIC, which corresponds to the previously validated cut-off values [21].

Finally, we registered all procedure-related complications, reoperations, and revision within the groups. This was done by local registration, where each site registered all complications during the 2 year follow-up period.

### Sample size

Our sample size was guided by identifying a 4-point difference in 2-year OKS between groups. Recommendations for between-group differences in OKS have ranged between 3 and 5 points historically [22,23], and we chose 4 points in our power calculation. The power calculation was based on a 2-sided t-test, and to obtain 90% power with a 2-sided significance level of 5%, and an expected standard deviation of 10, a total of 266 patients were required. To allow a 15% dropout before the 2-year-follow-up, we proceeded with a sample size of 314 patients.

### Statistics

Continuous variables were presented with a group mean and standard deviation (SD) or 95% confidence intervals (CI). Categorical data was presented as proportions with percentages (%) of total group size. Differences between groups were presented with 95% CI. The difference in our primary outcome, OKS 2 years post-surgery, was tested with a multiple linear regression model, which adjusted for group differences in age, sex, BMI, and baseline PRO score. All secondary PRO data, including TWA, was analyzed using the same approach. To ensure that the regression model’s assumptions were met, we checked for linearity, variance homogeneity, and normality in the residuals in a residual vs fitted values plot and a normal Q–Q plot. The assumptions were met in all models.

Missing data was excluded from the model in the 2 year post-surgery analysis. Categorical data was tested for association between group allocation and proportional distributions using a Fisher’s exact test. All analysis was conducted in RStudio (RStudio 2024.04.2; R Foundation for Statistical Computing, Vienna, Austria).

### Ethics, registration, use of AI, funding, and disclosures

We obtained study approval from the Regional Committee on Health Research Ethics (H-16027757) on August 4, 2016, which was prior to study initiation. Our protocol was registered at clinicaltrials.gov (ID: NCT03073941). AI tools were not used.

All participating institutions received funding from Zimmer Biomet (Warsaw, IN, USA) to support this work. KRLM received travel expenses from Zimmer Biomet to present this work at EFORT 2023. The institution of CV has received travel expenses from Stryker with no relation to this work. KG and AT have received or may receive payments or benefits from Zimmer Biomet related to this work. ICMJE disclosure forms are available on the article page, doi: 10.2340/17453674.2025.43004

## Results

**Figure 1:**
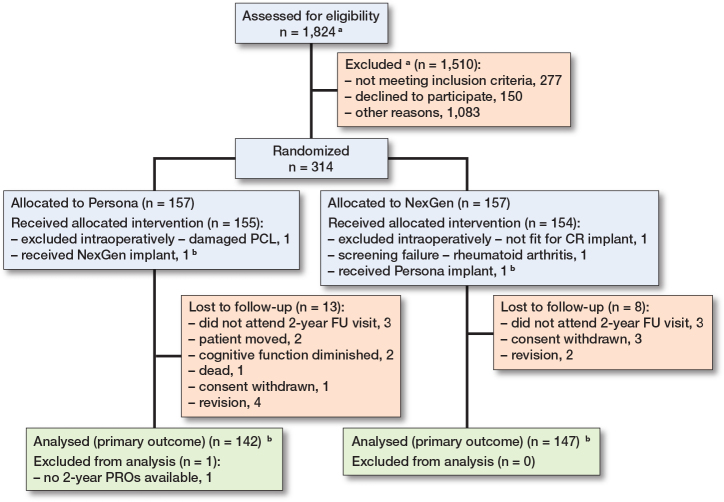
CONSORT flow diagram showing patient flow for primary outcome measurement through follow-up visits. ^a^ No screening list was available from 1 center (Næstved). ^b^ Patients with wrong treatment were analyzed with an intention-to-treat approach.

1 patient (NexGen) was excluded after randomization, and 2 patients were excluded intraoperatively (1 Persona, 1 NexGen), leaving 311 patients to be included in the analysis (Figure 1). Of the 311 patients, 2 patients (1 Persona, 1 NexGen) received the opposite implant to their randomization. We analyzed these 2 cases with an intention-to-treat approach. 289 patients (92%) were available for primary outcome analysis (Figure 1, Table 1).

**Table 1:** Baseline characteristics of the patients treated in the Persona and NexGen group. Mean values are presented with standard deviation (SD). Proportion of patients are presented with percentage of total group size (%)

Factor	Persona (n = 156)	NexGen (n = 155)
Age at surgery (SD)	69 (8)	68 (9)
Female sex, n (%)	99 (64)	90 (58)
BMI (SD)	30 (6)	31 (5)
Kellgren–Lawrence, n (%) ^a^		
2	6 (3.8)	10 (6.5)
3	57 (37)	48 (31)
4	92 (59)	96 (62)
Oxford Knee Score (SD) ^b^	22 (7)	22 (7)
Oxford Knee Score – APQ (SD) ^b^	16 (20)	15 (19)
Forgotten Joint Score (SD) ^b^	14 (14)	14 (13)
EQ-5D-3L Index (SD) ^b^	0.61 (0.18)	0.61 (0.19)
EQ-5D-3L VAS (SD) ^b^	62 (20)	64 (20)

APQ = Activity and Participation Questionnaire

a 2 patients (1 Persona, 1 NexGen) had no preoperative radiographs.

b 1 patient (NexGen) had no baseline PROs.

### Primary outcome

**Figure 2:**
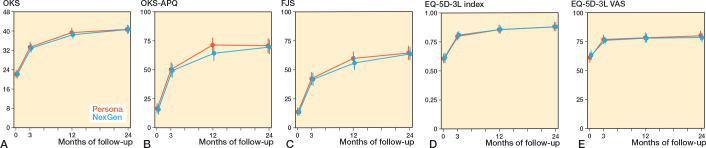
Mean with 95% confidence intervals patient reported outcomes (PROs) pre-surgery, 3 months, 1 year, and 2 years post-surgery. Persona group is red, NexGen group is blue. A is Oxford Knee Score, B is Oxford Knee Score – Activity and Participation Questionnaire, C is Forgotten Joint Score, D is EQ-5D-3L, and E is EQ-5D-3L VAS.

We found no statistically significant difference in the primary outcome, OKS, between the groups 2 years post-surgery. Unadjusted mean OKS was 40.7 (CI 39.5–41.9) and 40.8 (CI 39.7–41.9) in the Persona and NexGen groups respectively (Figure 2A, Table 2). Difference in adjusted mean OKS was 0.1 (CI –1.4 to 1.7; Table 2).

**Table 2:** Unadjusted means of patient reported outcome (PROs) 2 years post-surgery with 95% confidence intervals (CI). The difference in means between groups adjusted for differences in age, sex, BMI and baseline PRO score is presented

PROs	Persona mean (CI) (n = 142)	NexGen mean (CI) (n = 147)	Adjusted mean difference ^a^ (CI)	P value
Oxford Knee Score	40.7 (39.5–41.9)	40.8 (39.7–41.9)	0.1 (–1.4 to 1.7)	0.9
Oxford Knee Score – APQ	70.5 (65.3–75.7)	69.4 (64.3–74.5)	0.1 (–6.9 to 7.0)	1.0
Forgotten Joint Score	65.0 (60.2–69.8)	64.3 (59.9–68.7)	0.3 (–6.0 to 6.7)	0.9
EQ-5D-3L Index	0.89 (0.88–0.89)	0.88 (0.88–0.89)	0 (–0.04 to 0.04)	1.0
EQ-5D-3L VAS	81 (78–84)	79 (76–82)	–2.4 (–6.0 to 1.3)	0.2

APQ = Activity and Participation Questionnaire

a Difference in mean when adjusted for age, sex, BMI, and baseline score. 1 patient (NexGen) had no baseline PROs. Model is run with baseline value missing.

### Secondary outcomes

There were no differences in adjusted means between the groups in OKS-APQ, FJS, EQ-5D-3L Index score or EQ-5D-3L VAS 2 years post-surgery (Table 2). Additionally, adjusted mean in TWA of all PROs was not different between the groups at the first 2 years post-surgery (Table 3). All PRO score progressions are available in Figure 2.

**Table 3:** Time-weighted average (TWA) of patient-reported outcomes (PROs) over the entire observation period (from pre-surgery to 2 years post-surgery or dropout). The unadjusted TWA are presented with 95% confidence interval (CI). Mean differences between groups are presented when adjusted for differences in age, sex, BMI, and baseline PRO score

PROs	Persona TWA (CI) (n = 155) ^a^	NexGen TWA (CI) (n = 154) ^a^	Adjusted mean difference ^b^ (CI)	P value
Oxford Knee Score	36.6 (35.5–37.7)	36.4 (35.4–37.4)	– 0.1 (–1.5 to 1.2)	0.9
Oxford Knee Score – APQ	60.6 (56.4–64.8)	57.9 (53.8–62.0)	–1.9 (–7.5 to 3.7)	0.5
Forgotten Joint Score	52.7 (48.8–56.6)	51.5 (47.9–55.1)	–0.6 (–5.7 to 4.5)	0.8
EQ-5D-3L Index	0.83 (0.81–0.85)	0.83 (0.81–0.85)	0.01 (–0.02 to 0.03)	0.7
EQ-5D-3L VAS	78 (75–80)	77 (75–79)	–1.1 (–3.9 to 1.7)	0.4

APQ = Activity and Participation Questionnaire

a 2 patients (1 Persona, 1 NexGen) had no postoperative PROs at any follow-up, which is why TWA could not be calculated for these patients. 1 further patient (NexGen) had too many missing values in FJS, which is why n(FJS) = 153 for the NexGen-group.

b Difference in mean when adjusted for age, sex, BMI, and baseline score. 1 patient (NexGen) had no baseline value recorded. Model is run with baseline value missing.

Responder analysis on OKS showed no statistically significant association between group allocation and proportion of patients meeting criteria of PASS at any follow-up (Table 4). Additionally, no significant association between group allocation and proportions of patients with MIC in OKS or FJS was found at 1 year post-surgery (Table 4).

**Table 4:** Responder criteria analysis for the Oxford Knee Score (OKS). Number of patients reaching patient acceptable symptom state (PASS) is evaluated 3 months, 1 year, and 2 years post-surgery. Number of patients having a minimal important change (MIC) is evaluated 1 year post-surgery for OKS and the Forgotten Joint Score (FJS). Values are ratios and proportion in percentages

Responder criteria	Persona (%)	NexGen (%)	P value ^a^
PASS			
3 months (OKS ≥ 27)	130 of 154 (84)	121 of 151 (80)	0.4
1 year (OKS ≥ 30)	132 of 149 (89)	132 of 151 (87)	0.9
2 years (OKS ≥ 30)	130 of 142 (92)	138 of 147 (94)	0.5
MIC 1 year			
ΔOKS ≥ 8	133 of 149 (89)	125 of 150 (83)	0.2
ΔFJS ≥ 14	130 of 149 (87)	124 of 150 (83)	0.3

a Fisher’s exact test.

### Complications

We found no difference in procedure-related complications during the 2-year period with 7 of 156 (4.5 %) in the Persona group compared with 10 of 155 (6.5 %) in the NexGen group (Table 5) (P = 0.5). When sub-analyzing revision of any component, 4 of 156 (2.6%) were revised in the Persona group and 2 of 155 (1.3%) in the NexGen group (P = 0.7).

**Table 5:** Number of procedure-related complications and outcome of the event 2 years post-surgery. Association between group allocation and number of patients with a procedure-related complication is tested with a Fisher’s exact test

Complication	Number of events		Outcome/P value
	Persona n = 156	NexGen n = 155	
Flexion contracture and extension defect	1		Revision—all components
Tibial baseplate loosening		1	Revision—all components
Late deep infection		1	Revision—all components
Tibio-femoral instability	1		Revision—liner
Medial/lateral instability	1		Revision—liner
Dislocated liner	1		Revision—liner
Parapatellar exostosis	1		Reoperation to affected knee
Knee pain from inflamed synovium		1	Reoperation to affected knee
Patellar fracture after fall		1	Reoperation to affected knee
Stiff knee	1	4	Manipulation (brisement force)
Postoperative DVT	1	2	DVT treatment
Patients with AEs (any cause), n (%)	7 (4.5)	10 (6.5)	P = 0.5
Patients with revision (any component)	4 (2.6)	2 (1.3)	P = 0.7

DVT = deep vein thrombosis.

## Discussion

The aim of this RCT was to compare a new-generation TKA system with an optimized anatomical design, on PRO and complications, directly with its predecessor, which is a clinically proven system. We showed that there were no differences between the implants.

The development of more personalized, asymmetrical tibial components has led to superior outcomes in implant positioning aspects, such as bone coverage, overhang, and malrotation [24]. However, we were not able to show that the placement benefits of an asymmetric (Persona) component translate into improved short-term patient-perceived effects, when compared with a symmetric (NexGen) tibial component.

In our study, our primary outcome (OKS 2 years post-surgery) examines knee function and pain between the treatment groups. In both groups, OKS improved from pre-surgery to 2 years post-surgery in line with previous studies investigating OKS after TKA [9,25,26]. This indicates that the patients were experiencing improvements regardless of whether they were treated with a Persona or NexGen TKA. We believe this to be an important finding when considering the clinical performance of the implant.

Similar to the primary outcome, we were not able to demonstrate a difference in any secondary outcomes, but we did find clinically relevant improvements from baseline to 2 years post-surgery in both groups that were comparable to previous studies investigating the effectiveness of TKA, using the same outcomes [26-29]. This is also the case when applying the responder criteria of PASS and MIC, with a high proportion of patients achieving PASS and MIC in both groups. We also included a TWA analysis of all PROs collected. The TWA of a PRO is to be interpreted as the mean score most likely observed at any point in time within the observation period [19]. We believe this makes it an important aspect of reporting PROs, as we consider every day in the life of patient after TKA to be equally important, not just the result at a later stage post-surgery.

Our findings are in line with a recent study by Irmola et al., who performed an RCT comparing the clinical outcomes of treatment with the Persona, Nexgen, and P.F.C. (DePuy, Warsaw, IN, USA) implants 2 years post-surgery [26]. Applying numerous PROs, including OKS and FJS, the authors presented comparable scores to ours, with no clinically relevant difference between the groups. Their results, as well as the results of our study, could suggest that the difference in treatment outcome, should there be any, goes beyond what patients can report in PROs 2 years post-surgery. We suggest future studies to compare kinematic differences, measured with objective measurement tools, as a function of different implant options. This is especially relevant if the suggested differences between the compared implants produce a further balanced knee joint, due to more sizing options, after TKA.

The number of procedure-related complications was also comparable 2 years post-surgery (Table 5). Revision rates for any-cause revision during the first 2 years post-surgery were lower in both groups, when compared with the national average (Persona: 2.6%, NexGen: 1.3%, vs national average: 2.7%) [30], indicating that the safety of the implants used in our study was not a concern at an early stage. Longer term implant survival of the Persona and NexGen has been demonstrated in other Nordic registries. While no survival data on the specific implants is available in the Danish registry, the Norwegian registry presents a 3-year survival rate of the Persona as 98.7%, compared with NexGen at 98.1% [31]. Contrary to the Norwegian results, Swedish registry data shows a significantly higher hazard ratio for all-cause revision between 2014 and 2023, when Persona was directly compared with the NexGen implant (HR 1.60, CI 1.31–1.95) [32]. However, the later introduction of the Persona could lead to survivor bias, as many of the NexGen implants were possibly implanted well before the period accounted for, which is why the early failures in this group could have occurred before the comparison period had begun. Further, it is worth noting that substantially fewer Persona cases are reported on, both in Norway and in Sweden. As such, the combined registry results from Nordic countries cannot yet provide a clear conclusion on the revision rate, when comparing Persona with the NexGen TKA system. Therefore, we believe that further well-powered studies with longer follow-up periods and/or registry data should establish the long-term survivorship and performance, especially for the Persona TKA.

### Strengths

We enrolled 314 patients from 5 different centers nationally, while 92% were available for follow-up after 2 years, which limits any possible bias that could arise from missing data. Furthermore, patients remained blinded until primary outcome was collected, which would limit any ascertainment bias that could arise from patients’ attitude towards either a new and exciting or a well-proven treatment. Primary and secondary outcomes include well-validated questionnaires that examine important aspects of patient-perceived treatment quality after TKA, such as pain and knee function, which is why we believe that our study has provided solid documentation of comparable treatment quality, in aspects that matter greatly to the patients, with the studied implant systems.

### Limitations

First, our statistical power-calculation was based on finding differences in the OKS, which left us underpowered to detect any possible differences in procedure-related complications. Reoperations and revisions are often more frequent after the first couple of years post-surgery, and because of the relatively rare occurrence of complications a larger sample size and longer follow-up would be required to examine a possible difference in, e.g., implant survival.

Second, we performed this study during the introduction of the Persona knee, meaning that surgeons’ experience with this particular implant was limited. It is documented that the introduction of the Persona did increase surgical time and blood loss when introduced [6], indicating a learning curve when introducing new implants should be expected. However, the same study showed no differences in the number of alignment outliers, which could have caused poor clinical outcome and ultimately led to revision. As we found comparable high scores in, e.g., OKS, we believe surgeons’ possible limited experience with the implants before the study had a limited influence on our results, if any.

Finally, this study was carried out without a pre-specified and published statistical analysis plan. However, a protocol was registered at clinicaltrials.gov, where our primary outcome and desired sample size are described. To provide further transparency, we have described the power calculation and shown that it corresponds to the statistical test performed in our analysis for this paper.

### Conclusion

In this RCT, we found a non-significant mean difference in OKS between patients treated with Persona or NexGen TKAs well below limits of clinical relevance, 2 years post-surgery. Furthermore, no clinically relevant or significant difference in any patient-reported outcome 2 years post-surgery, time-weighted averages of patient-reported outcomes, proportion of patients with PASS or MIC, or number of procedure-related complications were found during the study period.

*In perspective,* further studies should document the long-term safety and clinical outcomes of the Persona Total Knee System.

## Notes

Authorships were granted according to ICMJE standards and contributions are expressed according to the CRedIT author statement. KRLM: investigation, data curation, formal analysis, validation, visualization, writing—original draft, writing—review and editing. LHI: investigation, formal analysis, validation, writing—review and editing. CV: investigation, project administration, validation, writing—review and editing. AO, AK, and HS: conceptualization, methodology, investigation, project administration, validation, writing—review and editing. KG and AT: conceptualization, methodology, supervision, investigation, project administration, validation, writing—review and editing.

Handling co-editors: Keijo Mäkelä and Philippe Wagner

*Acta* thanks Ove Nord Furnes and Martijn Schotanus for help with peer review of this manuscript.
